# Association between epilepsy and psychiatric disorders in adults with intellectual disabilities: systematic review and meta-analysis

**DOI:** 10.1192/bjo.2021.55

**Published:** 2021-05-03

**Authors:** Basma Akrout Brizard, Bharati Limbu, Carolina Baeza-Velasco, Shoumitro Deb

**Affiliations:** Université de Paris, Laboratory of Psychopathology and Health Processes, F-92100 Boulogne Billancourt, France; Department of Brain Sciences, Faculty of Medicine, Imperial College London, UK; Laboratory of Psychopathology and Health Processes, Université de Paris, France; and Department of Emergency Psychiatry and Acute Care, CHU Montpellier, France; Division of Psychiatry, Department of Brain Sciences, Faculty of Medicine, Imperial College London, UK

**Keywords:** Intellectual disabilities, epilepsy, psychiatric disorders, systematic review, meta-analysis

## Abstract

**Background:**

Psychiatric disorders, such as depression and anxiety, are commonly associated with epilepsy in the general population, but the relationship between psychiatric disorders and epilepsy among adults with intellectual disabilities is unclear.

**Aims:**

To conduct a systematic review and meta-analysis to assess whether epilepsy is associated with an increased rate of psychiatric disorders in adults with intellectual disabilities.

**Method:**

We included literature published between 1985 and 2020 from four databases, and hand-searched six relevant journals. We assessed risk of bias by using SIGN 50 and the Cochrane risk of bias tool. Several meta-analyses were carried out.

**Results:**

We included 29 papers involving data on 9594 adults with intellectual disabilities, 3180 of whom had epilepsy and 6414 did not. Of the 11 controlled studies that compared the overall rate of psychiatric disorders between the epilepsy and non-epilepsy groups, seven did not show any significant inter-group difference. Meta-analysis was possible on pooled data from seven controlled studies, which did not show any significant inter-group difference in the overall rate of psychiatric disorders. The rates of psychotic disorders, depressive disorders and anxiety disorders were significantly higher in the non-epilepsy control groups compared with the epilepsy group, with effect sizes of 0.29, 0.47 and 0.58, respectively. Epilepsy-related factors did not show any definite association with psychiatric disorders.

**Conclusions:**

It is difficult to pool data from such heterogeneous studies and draw any definitive conclusion because most studies lacked an appropriately matched control group, which will be required for future studies.

Intellectual disabilities are a group of aetiologically diverse conditions originating during the developmental period, characterised by significantly below-average intellectual functioning and adaptive behaviour that are approximately two or more standard deviations below the mean, based on appropriately normed, individually administered standardised tests.^1^

## Epilepsy in adults with intellectual disabilies

The prevalence of epilepsy is much higher in adults with intellectual disabilities (>25%) than in the general population (1%).^2^ Compared with the general population, epilepsy among adults with intellectual disabilities is not only more prevalent, but often manifests as multiple seizure types, starts at an early age, is of longer duration and is more treatment-resistant (around 30% in the general population compared with >70% in people with intellectual disabilities).^3^ Diagnosing epilepsy and seizure types can be difficult in this population. For example, stereotypy, cardiac syncope and non-epileptic attack disorders may all mimic epileptic seizures. On the other hand, absence and partial seizures may be particularly challenging to detect in this population.^4^ Thus, both a false positive and a false negative diagnosis are possible.^4^ Also, people with intellectual disabilities are more prone to die from sudden unexpected death in epilepsy.^5^

## Epilepsy and psychiatric disorders in the general population

Studies in the general population found an increased rate of psychiatric disorders in adults with epilepsy. For example, the prevalence of mood and anxiety disorders is reported to be 20–30%, whereas psychoses are estimated to be 2–7% in the general population with epilepsy.^6^ These figures are higher than the point prevalence of 17% of mood and anxiety disorders and 1–2% of psychoses observed among the general population who do not have a diagnosis of epilepsy.^7^ The affective disorders tend to be present at all stages of epilepsy, whereas psychosis is particularly prevalent in the post-ictal phase.

## Psychiatric disorders in intellectual disabilities

If problem (challenging) behaviour and autism spectrum disorder (ASD) are included, the overall rate of mental ill health seems higher in adults with intellectual disabilities (40.9%)^8^ than in the general population (16%).^9^ However, excluding the diagnosis of ASD and problem behaviour, the rate of overall functional psychiatric disorders (14.5%)^8,10^ is similar to that in the general population (16%).^9^ The point prevalence of psychosis including schizophrenia is significantly higher in adults with intellectual disabilities (3.4–4.4%)^10–12^ compared with adults without intellectual disabilities (1%).^9,13^ Although depressive symptoms are reported in 16.5% of adults with intellectual disabilities, the rate of major depressive disorder seems similar in both adults with intellectual disabilities (2.2–8%)^8,10,12^ and adults without intellectual disabilities (2.1%).^9^ The rate of anxiety disorder seems higher in adults with intellectual disabilities (14%)^12^ than in the general population (10%).^13^ However, diagnosis of psychiatric disorders could be difficult in many adults with intellectual disabilities, particularly those who have a severe and profound intellectual disability and cannot communicate their thoughts and feelings to others.^14^ Therefore, both a false positive and a false negative diagnosis are possible.

## The need for this systematic review

The question of whether there is an association between epilepsy and psychiatric disorders in adults with intellectual disabilities remains unanswered. We found only one systematic review of neuropsychiatric conditions in people with intellectual disabilities^15^ that included 15 studies, but only two of these are specifically on psychiatric disorders *per se*, and the rest are mostly on problem behaviour. Also, the previous review included participants of all ages and did not present data separately on adults, which is the focus of the current review. We have already published a systematic review with meta-analysis specifically on the relationship between epilepsy and problem behaviour in adults with intellectual disabilities.^16^ As there is currently no published systematic review and meta-analysis available specifically on the association between psychiatric disorders and epilepsy in adults with intellectual disabilities, we have carried out a systematic review and meta-analysis on studies that explored this relationship. We have concentrated on studies of adults with intellectual disabilities only, as the issues concerning children with intellectual disabilities are different from those of adults.

## Method

### Search strategy

Protocol and search strategy were based on the International Prospective Register of Systematic Reviews (PROSPERO) guidelines^17^ and the Preferred Reporting Items for Systematic Review and Meta-Analysis Protocols (PRISMA-P) checklist.^18^ The study was registered with PROSPERO, under registration number CRD42020178083.

Four electronic databases were searched for relevant studies: EMBASE, PsycINFO, PubMed and DARE. The electronic search focused on articles published in English and French, between 1 January 1985 and 31 May 2020. Hand-searching for relevant articles was carried out in the past 10 years of issues, from January 2000 to June 2020, in the following journals: *Seizure*, *Epilepsia*, *Epilepsy & Behavior*, *Journal of Intellectual Disability Research*, *Journal of Applied Research in Intellectual Disabilities* and *Research in Developmental Disabilities*. Only quantitative studies were searched.

### Search terms

Each database was searched using terms for intellectual disability, epilepsy and psychiatric disorders.

Terms for intellectual disabilities were: ‘Intellectual disability’ OR ‘Learning disability’ OR ‘Learning disorder’ OR ‘Learning difficulties’ OR ‘Mental disabilities’ OR ‘Neurodevelopmental disorders’ OR ‘NDD’ OR ‘ID’ OR ‘LD’ OR ‘Mental retardation’ OR ‘Mental handicap’ OR ‘Mental deficiency’.

The following search terms were used to cover psychiatric disorders: ‘Psychiatric illness’ OR ‘Psychiatric disorders’ OR ‘Schizophrenia’ OR ‘Psychosis’ OR ‘Depression’ OR ‘Major depressive disorder’ OR ‘Bipolar disorder’ OR ‘Mania’ OR ‘Hypomania’ OR ‘Anxiety disorders’ OR ‘Anxiety’ OR ‘Obsessive-Compulsive Disorder’ OR ‘OCD’ OR ‘Phobia’ OR ‘Phobic disorders’ OR ‘Personality disorder’ OR ‘Dementia’.

Terms for epilepsy were: ‘Epilepsy’ OR ‘Epilepsy syndrome’ OR ‘Seizures’ OR ‘Seizure disorders’ OR ‘Epileptic seizures’ OR ‘AED’ OR ‘Anti-epileptic drugs’.

### Criteria for selecting studies for this review

A list of eligibility criteria, based on PROSPERO guidelines^17^ and *Cochrane Handbook for Systematic Reviews of Interventions*,^19^ was adapted. The eligibility criteria for the review were (a) an epilepsy and intellectual disabilities group compared with a non-epilepsy group of adults with intellectual disabilities alone (if the study did not have a non-epilepsy control group, the study had to provide information on psychiatric disorders, epilepsy-related factors and anti-epileptic drug regimen); (b) all participants had intellectual disabilities; (c) all participants were defined as adults by the authors and (d) a minimum sample size of ten participants.

### Types of studies

This review included studies with different designs. Both randomised and non-randomised studies, and both controlled and non-controlled observational or cross-sectional studies, were included. Controlled studies with both matched and non-matched control groups were included.

We included studies that compared the overall rate of psychiatric disorders, as well as different types of psychiatric disorders in adults with intellectual disabilities and epilepsy, with a group of adults with intellectual disabilities without epilepsy within the same cohort.

We also included studies that included participants with intellectual disabilities and epilepsy, but no participants with intellectual disabilities without epilepsy. These studies allowed assessment of the association of psychiatric disorders with epilepsy-related variables.

Studies were included regardless of the method used to assess the rate of any psychiatric disorders and specific psychiatric disorders, such as affective disorders, psychosis, anxiety disorders, personality disorders and dementia. In that, studies reporting data with standardised assessment tools, case notes, symptom checklists and semi-structured interviews administered by clinicians or trained professionals were included.

### Types of participants

All participants were adults aged ≥16 years, had intellectual disabilities (all levels of severity) and had various types of psychiatric disorders. This review focuses on data on psychiatric disorders and does not present data on problem behaviour, as a separate systematic review has been published recently on the association between epilepsy and challenging (problem) behaviour in adults with intellectual disabilities.^16^

Ethical approval was not required for this study because no individual patient-related data were collected or analysed.

### Secondary outcome

Comparison between epilepsy and non-epilepsy groups was carried out for different types of psychiatric disorders (psychotic disorders, depressive disorders, anxiety disorders, personality disorders and dementia). To identify the role of different epilepsy-related factors in the development of psychiatric disorders, data on subgroup comparisons according to types of seizures, frequency of seizures and drug regimen (e.g. polypharmacy versus mono-pharmacy) were collected.

### Selection process

After completion of each database search, references were recorded on Zotero reference management software version 5.0.77 for Windows (Corporation for Digital Scholarship, George Mason University, US; see https://www.zotero.org/download/).^20^ Titles were searched for key terms. Non-human studies, studies involving children and people without intellectual disabilities were removed. Duplicates were identified by Zotero, and removed manually by the first author (B.A.B.). Independent screening of the remaining abstracts was carried out by B.A.B. and B.L., using pre-piloted eligibility criteria. The review authors were blind to each other's scores. Discrepancies identified were reviewed and discussed until resolution of differences by consensus. Full texts were gathered for the studies that met the inclusion criteria or the ones marked as uncertain. The full texts were then reviewed and assessed by both reviewers (B.A.B. and B.L.), using the same eligibility checklist that was used for screening abstracts.

**Fig. 1:**
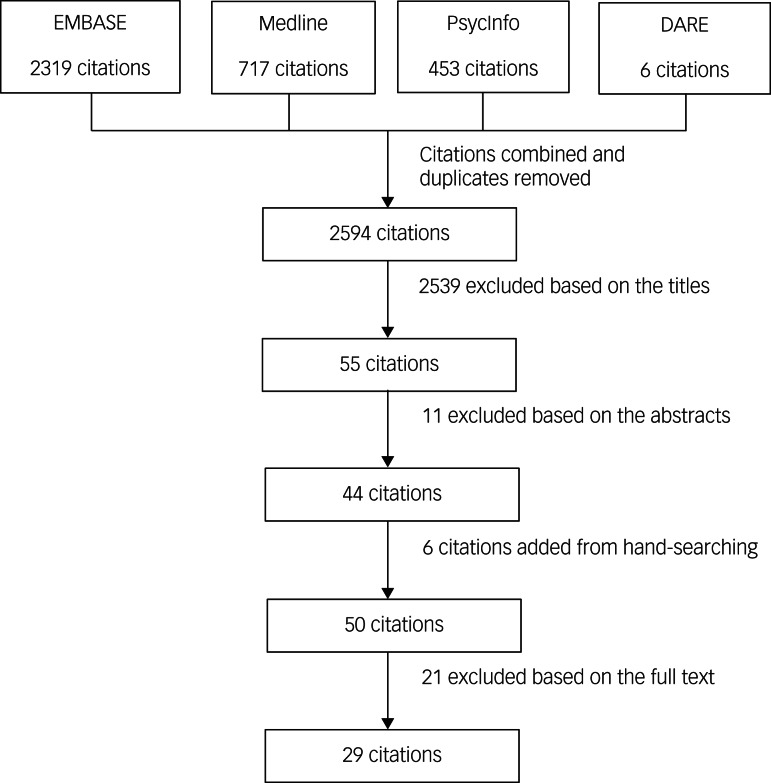
Preferred Reporting Items for Systematic Review and Meta-Analysis flow chart of the study selection process.

The selection process is reported in a PRISMA flow diagram (see Fig. 1). It was not necessary for a third review author (S.D.) to arbitrate.

### Data extraction

Data from studies meeting eligibility criteria were extracted by both reviewers (B.A.B. and B.L.), using a standardised data extraction proforma adapted from the *Cochrane Handbook for Systematic Reviews of Interventions* (see Supplementary Appendix 1 available at https://doi.org/10.1192/bjo.2021.55).^21^ The third review author (S.D.) checked the collected data for accuracy. Data extraction started on 8 July 2020. We have presented data separately on the more recently published studies since 2010 because definitions of epilepsy, intellectual disabilities and psychiatric disorders have changed in the past decade.

### Meta-analysis

We used RevMan version 5.3 for Windows 10 (The Cochrane collaboration, London, UK; see https://training.cochrane.org/online-learning/core-software-cochrane-reviews/revman/revman-5-download) meta-analysis software for the random-effects model. The random-effects model was used because of differing study designs.

As most studies presented prevalence rates among different groups but a few others presented the mean and s.d., we only included studies presenting prevalence rates of psychiatric disorders in each group (epilepsy versus non-epilepsy group) for meta-analysis. A forest plot was constructed for comparison, for which we reported the pooled odds ratio, 95% confidence interval and a *P*-value. The statistical significance level was set at *P* < 0.05. Heterogeneity was tested with *χ*^2^ and *I*^2^ values. Heterogeneity was considered minimal when under 40% and substantial when over 50%, according to guidelines from the *Cochrane Handbook for Systematic Reviews of Interventions*.^21^ Sensitivity analysis was carried out when heterogeneity was considered substantial.

### Risk of bias assessment

The Scottish Intercollegiate Guideline Network (SIGN 50) checklist^22^ was used to assess the quality of the 29 included studies. Additionally, the risk of bias for 25 controlled studies was assessed by the Cochrane risk of bias tool.^23^

### Confidence in the cumulative estimate

We assessed publication bias with a funnel plot and Egger's test, and assessed the included studies for consistency and precision. We excluded any studies deemed of low quality. We assessed the quality of the systematic review by using A Measurement Tool to Assess Systematic Reviews 2nd edition (AMSTAR-2) criteria (see Supplementary Appendix 2).^24^

## Results

### Search findings

A total of 2594 articles were screened based on titles. After screening with the eligibility criteria, 2539 articles were excluded. The remaining 55 abstracts were in English, and no studies in French were selected for abstract screening. We screened 55 abstracts with the eligibility criteria, and excluded 11 citations.

With an additional six citations detected through hand-searching and cross-referencing, a total of 50 full texts were screened with the eligibility criteria. Of these, 29 articles met the eligibility criteria and were ultimately included in the systematic review (see Fig. 1). A list of excluded articles with the reason for exclusion is presented in Supplementary Appendix 3.

### Included studies

Twenty-nine papers were included in our systematic review. Three articles published data on the same cohort but different outcome measures. Six studies included participants >16 years of age, three studies included participants >17 years of age and three stated that the participants were adults. We have included all these studies, as authors defined the study population as adults, and age cut-off for defining adulthood varies from country to country. for legal and administrative purposes. Of the 29 studies, 11 were controlled studies of comparison of the overall rate of psychiatric disorders (two of which were matched), and 14 were controlled studies that presented data on specific psychiatric disorders between epilepsy and non-epilepsy groups. Four studies included only participants with intellectual disabilities and epilepsy, and did not have a control group. Table 1 presents data from studies that compared data on the overall rate of psychiatric disorders in adults with intellectual disabilities and epilepsy and adults with intellectual disabilities who did not have epilepsy. In two studies,^25,26^ the groups were matched, and the number of participants remained the same in the epilepsy and non-epilepsy groups. In the remaining nine studies, the two groups of adults with intellectual disabilities with and without epilepsy were not matched. ^10,27–34^ In the unmatched studies, the prevalence of psychiatric disorders was collected in a larger sample of adults with intellectual disabilities, only a proportion of whom (around 27%) had epilepsy. The rates of psychiatric disorders were compared between adults with intellectual disabilities with and without epilepsy within the same sample, but the two groups were not matched.

**Table 1:** The rates of psychiatric disorders in adults with intellectual disabilities and with and without epilepsy

Reference	Sample, control group, study design	Age, years	Intellectual disability diagnosis	Epilepsy diagnosis	Measures used	Statistical analysis	Results
Matched control group							
Deb and Hunter, 1991^25^/UK	150 with intellectual disabilities and epilepsy matched with 150 with intellectual disabilities alone	20–77	Various psychometric tests (WAIS, WAIS-R, Raven's progressive matrices, Peabody Picture Vocabulary Test, Vineland Social Maturity Scale)	At least three epileptic seizures in 2 years, according to Gunn and Fenton's (1969)^48^ operational definition	Screening with PAA followed by PSE interview followed by a DSM-III-R diagnosis	*χ*^2^	The non-epilepsy group (31.33%) showed a significantly higher rate of psychiatric disorders than the epilepsy group (19.33%) (*χ*^2^ = 4.036, d.f. 1, *P* < 0.05); 32% of those with mild-to-moderate intellectual disabilities compared with 17% with severe intellectual disabilities had a psychiatric disorder (*P* < 0.01)
Matthews et al, 2008^26^/UK	55 with intellectual disabilities and epilepsy matched with 55 with intellectual disabilities alone	18–78	No diagnosis: keyword searches of computerised notes and records of medication	No diagnosis: keyword searches of computerised notes and records of medication	PIMRA	*χ*^2^	No significant inter-group difference in the proportion of participants meeting the PIMRA threshold level indicating a possible psychiatric disorder (54.5% for the epilepsy group compared with 44.4% for the non-epilepsy control group, *χ*^2^ = 1.11, *P* = 0.292)
Unmatched control group							
Cowley et al, 2004^28^/UK	752 with intellectual disabilities including 21% with epilepsy (63% with mild, 23% with moderate and 14% with severe intellectual disabilities)	Adults	IQ < 70, significant social impairment, and both conditions present from childhood	Case notes	ICD-10	Logistic regression	A statistically significant lower rate of psychopathology in the epilepsy group compared with the non-epilepsy control group (*P* < 0.01)
Deb et al, 2001^10^/UK	90 with intellectual disabilities including 21 with epilepsy (53.3% with mild and 46.7% with moderate intellectual disabilities)	16–64	Subjective assessment based on participant's level of communication skills and autonomy	Case notes	Screening with mini PAS-ADD interview followed by full PAS-ADD interview to make an ICD-10 diagnosis	*χ*^2^	No statistically significant difference in the rate of psychiatric disorders in epilepsy (5/21, 23.8%) and the non-epilepsy group (8/69, 11.6%). Psychiatric disorders were reported in 14.6% of adults with mild and 14.3% with moderate intellectual disabilities. The statistical significance was not reported
Espie et al, 2003^30^/UK	172 with intellectual disabilities and epilepsy compared with 127 with intellectual disabilities alone from a different study sample	Mean 35.5, s.d. 10.1	Vineland Adaptive Behaviour Scales combined with available clinical or psychometric reports	Neurologist's diagnosis, seizure diaries to estimate the frequency	PAS-ADD checklist	Regression analysis	No difference between the two samples in the rate of psychiatric caseness (33%)
Lund, 1985^32^/Denmark	302 with intellectual disabilities including 55 with epilepsy	> 20	ICD-8 criteria	Information about epilepsy and anti-epileptic treatment collected from case records, EEG and interviews with medical persons and parents	An author-devised psychiatric schedule	*χ*^2^	No statistically significant inter-group difference in the rate of psychiatric disorder between the lifetime epilepsy group (36.3%) and the non-epilepsy control group (26.3%). More severe intellectual disability was significantly associated with a higher rate of both epilepsy and psychiatric disorder (*P* < 0.05)
Mantry et al, 2008^34^/UK	186 with Down syndrome including 24 with epilepsy (41.4% with mild, 26.9% with moderate, 18.3% with severe and 13.4% with profound intellectual disabilities)	>16	Not mentioned	Not mentioned	Clinician's diagnosis	*χ*^2^	Of those who had a diagnosis of mental ill health, 29.2% had epilepsy and 22.2% did not have epilepsy, and this inter-group difference was not statistically significant (*P* = 0.472)
McGrother et al, 2006^33^/UK	2393 with intellectual disabilities including 620 with epilepsy	>20	ICD-10	Questionnaire based	Author-devised questionnaire	Logistic regression	People with epilepsy had a significantly higher rate of psychological symptoms compared with the non-epilepsy group (79.4% *v.* 74.2%, respectively; odds ratio 1.34, adjusted *P* = 0.034)

WAIS, Wechsler Adult Intelligence Scale, WAIS-R, Wechsler Adult Intelligence Scale – Revised; PAA, Profile of Abilities and Adjustment Schedule; PSE, Present State Examination; PIMRA, Psychopathology Instrument for Mentally Retarded Adults; PAS-ADD, Psychiatric Assessment Schedule for Adults with Developmental Disabilities; EEG, electroencephalogram.

Table 2 presents data on the rates of different types of psychiatric disorders with comparisons between epilepsy and non-epilepsy groups of adults with intellectual disabilities. Table 3 includes data on epilepsy-related factors associated with psychiatric disorders in adults with intellectual disabilities and epilepsy. Table 4 presents data separately on the studies published in the past decade (2010 onward).

**Table 2:** Types of psychiatric disorders in adults with intellectual disabilities and epilepsy

Reference	Study design	Sample, control group	Age, years	Intellectual disability diagnosis	Epilepsy diagnosis	Measures used	Statistical analysis	Results
Collacott, 1993^54^/UK	Observational study	35 with Down syndrome and epilepsy (including 5 with dementia) compared with 68 with Down syndrome only	Adults	Medical records	At least three seizures within 2 years	Case notes	*χ*^2^	A significant association between a clinical diagnosis of dementia and the presence of epilepsy in those whose seizure started after the age of 35 years (*χ*^2^ = 56.27, d.f. 2, *P* < 0.001)
Cooper et al, 2007^11^/UK	Observational study	1023 adults with intellectual disabilities including 349 with epilepsy (38.9% with mild, 24.2% with moderate, 18.9% with severe and 18% with profound intellectual disabilities)	Mean 43.9 (range 16–83)	Vineland Scale (Survey Form)	Not mentioned	PAS-ADD, PPS-LD	Multivariate analysis	Six of 349 (1.7%) participants in the epilepsy group, compared with 20 of 674 (3%) in the non-epilepsy group, had a diagnosis of psychosis (odds ratio 0.27, 95% CI 0.11–0.65, *β* = −1.31, *P* = 0.004)
Cowley et al, 2004^28^/UK	Observational study	752 with intellectual disabilities including 21% with epilepsy (63% with mild, 23% with moderate and 14% with severe intellectual disabilities)	adults	IQ < 70, significant social impairment and both conditions present from childhood	Not mentioned	Psychiatric diagnosis based on ICD-10 criteria	Logistic regression	A statistically significant lower rate of schizophrenia spectrum disorder in the epilepsy group compared with the non-epilepsy control group (*P* < 0.05)
Deb and Hunter, 1991^25^ /UK	Matched controlled study	150 with intellectual disabilities and epilepsy, matched with 150 with intellectual disabilities alone	20–77	See Table 1	See Table 1	Screening with PAA followed by PSE interview followed by a DSM-III-R diagnosis	*χ*^2^	A non-significantly higher rate of major depression in the non-epilepsy group (*n* = 4, 2.66%) than the epilepsy group (*n* = 1, 0.66%). A non-significantly higher rate of OCD in the non-epilepsy group (*n* = 4, 2.66%) than the epilepsy group (*n* = 2, 1.33%). A non-significantly higher rate of phobia in the non-epilepsy group (*n* = 10, 6.66%) than the epilepsy group (*n* = 5, 3.33%). A non-significantly marginally higher rate of dementia in the non-epilepsy group (*n* = 4, 2.66%) than the epilepsy group (*n* = 3, 2%). A non-significantly higher rate of schizophrenia and delusional disorders in the epilepsy group (*n* = 4, 2.66%) than the non-epilepsy group (*n* = 0). A statistically significant higher rate of bipolar disorder in the non-epilepsy group (*n* = 6, 4%) than the epilepsy group (*n* = 0). Psychotic disorders were reported in 15% of participants with mild-to-moderate intellectual disabilities and 5.6% of those with severe intellectual disabilities (*P* < 0.05)
Deb and Hunter, 1991^35^/UK	Matched controlled study	75 with mild-to-moderate intellectual disabilities and epilepsy, 75 with mild-to-moderate intellectual disabilities alone	20–77	Various psychometric tests (WAIS, WAIS-R, Raven's progressive matrices, Peabody Picture Vocabulary Test, Vineland Social Maturity Scale)	At least three epileptic seizures in 2 years, according to Gunn and Fenton's (1969)^48^ operational definition	SAP Schedule, T-L PBI	*χ*^2^	No statistically significant inter-group difference between the epilepsy and non-epilepsy groups in either the rate of personality disorder according to total SAP scale score (odds ratio 0.47, 95% CI 0.24–0.92) or aggressive personality type score. No significant inter-group difference according to the T-L PBI personality trait score
McCarron et al, 2005^55^/Ireland	Retrospective cohort study	124 with Down syndrome including 42 with epilepsy (35 with mild, 69.4% with moderate and 30.6% with severe intellectual disabilities)	>35	Not mentioned	Questionnaire	Case notes	*χ*^2^	Epilepsy is significantly more common in those with Alzheimer's disease compared with those without: 55.5% *v.* 11.4%, respectively (*χ*^2^ = 22.89, d.f. 1, *P* < 0.001)
McGrother et al, 2006^33^/UK	Population-based study	2393 with intellectual disabilities including 620 with epilepsy	>20	See Table 1	See Table 1	Author-devised interview	Logistic regression	The epilepsy group showed significantly higher rates of autistic traits compared with the non-epilepsy group (57.1% *v.* 41.6%, odds ratio 1.87, *P* < 0.0001). No statistically significant difference between epilepsy and non-epilepsy groups in anxiety symptoms (34.4% *v.* 38%, odds ratio 0.86, *P* = 0.459)
Pawar and Akuffo, 2008^57^/UK	Comparative survey	177 with intellectual disabilities including 53 with epilepsy, unmatched	>17	Not mentioned	Pre-formatted data collection sheet	Case notes	Descriptive statistics	When compared with the non-epilepsy controls, the epilepsy group showed lower rates of (a) depression (26% of non-epilepsy group *v.* 19% of epilepsy group), (b) anxiety disorders (19% *v.* 8%), (c) psychoses (19% *v.* 2%), (d) autism spectrum disorder (11% *v.* 9%), (e) bipolar disorder (6% *v.* 0%) and (f) personality disorder (5% *v.* 2%), but not dementia (8% *v.* 8%). The statistical significance of these inter-group differences is not reported. The epilepsy group showed a lower rate of mild (40%) and moderate intellectual disabilities (26%) compared with the non-epilepsy group (58% and 31%, respectively). However, the epilepsy group showed a higher rate of severe intellectual disability (30%) compared with the non-epilepsy group (11%). The statistical significance was not reported
Reid and Ballinger, 1987^49^/UK	Observational study	100 with intellectual disabilities including 25 with epilepsy, hospital in-patients, unmatched (34% with mild and 66% with moderate intellectual disabilities)	21–81	Mild (IQ range 50–70) or moderate (IQ range 35–49)	Three or more fits over the past 2 years or were still receiving anticonvulsant medication for previous epilepsy, according to Gunn and Fenton's (1969) operational definition^48^	SAP	*χ*^2^	No statistically significant inter-group difference in personality disorders between the epilepsy group (60%) and non-epilepsy group (54%)
Turkistani, 2004^59^/UK	Retrospective study	108 with intellectual disabilities and epilepsy versus 132 with intellectual disabilities alone, unmatched	Mean 40.3	Not mentioned	Active epilepsy: at least one seizure in the past 2 years.	Case notes, based on ICD-10 diagnosis	*χ*^2^	No significant difference in the rate of psychosis between the epilepsy group (6/108, 5.5%) and the non-epilepsy control group (21/132, 15.9%). The non-epilepsy control group showed a significantly higher rate of depression compared with the epilepsy group, at 25% (33/132) *v.* 6.5% (7/108), respectively (*χ*^2^ = 14.68, *P* = 0.001). There was a significant difference in the severity of intellectual disability between the epilepsy and non-epilepsy groups (*P* = 0.003), with the epilepsy group showing lower rates of mild (9.25% *v.* 12.87%) and moderate intellectual disabilities (36.11% *v.* 53.03%), but higher rates of severe (38.88% *v.* 29.54%) and profound intellectual disabilities (15.74% *v.* 4.52%)
Tyrrell et al, 1996^51^/Ireland	Cross sectional study	76 females with Down syndrome including 13 with epilepsy (6/76 with dementia)	>35 (mean 47.3, s.d. 8.8)	Moderate (IQ range 35–50), severe (IQ range 20–35)	ILAE criteria (1981)	TSI, DSMSE	*χ*^2^	Epilepsy was significantly more common in adults with dementia compared with those who did not have dementia (*P* = 0.005)
Tyrrell et al, 2001^44^/Ireland	Cross sectional study	285 with Down syndrome including 58 with epilepsy (38 with dementia)	>35	ICD-10 criteria and IQ evaluation (mild <70, moderate <50, severe <35, profound <20)	ILAE criteria (1981)	TSI, DSMSE	*χ*^2^	Epilepsy was significantly more common in people with dementia compared with those who did not have dementia (*χ*^2^ = 55.5, d.f. 1, *P* < 0.0001)

PAS-ADD, Psychiatric Assessment Schedule for Adults with Developmental Disabilities; PPS-LD, Psychopathology Schedule for Adults with Learning Disabilities; PAA, Profile of Abilities and Adjustment Schedule; PSE, Present State Examination; OCD, obsessive–compulsive disorder; WAIS, Wechsler Adult Intelligence Scale, WAIS-R, Wechsler Adult Intelligence Scale – Revised; SAP, Standardized Assessment of Personality; T-L PBI, Temporal Lobe Personality Behaviour Inventory; ILAE, International League Against Epilepsy; TSI, Test for Severe Impairment; DSMSE, Down Syndrome Mental Status Examination.

**Table 3:** Psychiatric disorders according to different epilepsy variables

Reference	Study design	Sample, control group	Age, years	Intellectual disability diagnosis	Epilepsy diagnosis	Measures used	Statistical analysis	Results
Deb and Hunter, 1991^25^/UK	Matched controlled study	150 with intellectual disabilities and epilepsy and a matched control group of 150 with intellectual disabilities alone	20–77	See Table 1	See Table 1	DSM-III-R	Wilcoxon	Compared with a matched control group of intellectual disabilities alone, epilepsy group showed a significantly lower rate of psychiatric disorders in (a) active epilepsy group (seizure within the past 12 months) (*χ*^2^ = 5.048, d.f. 1, *P* = 0.025), (b) those who had epilepsy for >19 years (*χ*^2^ = 19.057, d.f. 1, *P* = 0.000), (c) those who received polypharmacy of anti-epileptic medication (*χ*^2^ = 6.976, d.f. 1, *P* = 0.008) and (d) those who had frequent seizures (*χ*^2^ = 5.149, d.f. 1, *P* = 0.023). However, a significantly higher rate of psychiatric disorder was reported in people suffering from epilepsy for <19 years (*χ*^2^ = 6.649, d.f. 1, *P* = 0.009)
Deb and Hunter, 1991^35^/UK	Matched controlled study	75 with mild-to-moderate intellectual disabilities and epilepsy; 75 with mild-to-moderate intellectual disabilities alone	20–77	See Table 2	See Table 2	SAP Schedule, T-L PBI	*χ*^2^	Compared with non-epilepsy controls, the epilepsy group showed a higher rate of temporal lobe personality disorder among (a) those who were living in the community than those who were hospital in-patients (*χ*^2^ = 6.825, d.f. 1, *P* < 0.01), (b) those who received polypharmacy of anti-epileptic medications than those who received mono-pharmacy (*χ*^2^ = 5.877, d.f. 1, *P* < 0.02) and (c) those who had seizures in the past 12 months than those who did not (*χ*^2^ = 4.444, d.f. 1, *P* < 0.05)
Deb and Joyce, 1999^50^/UK	Retrospective study	143 with intellectual disabilities and epilepsy (12.6% with mild, 16.1% with moderate and 55.9% with severe intellectual disabilities)	20–83	IQ (mild 70–50, moderate 49–35, severe < 35)	ILAE criteria (1981)	ICD-10 diagnosis based on case notes	*χ*^2^	The rate of psychiatric illness was significantly higher in those whose EEG showed epileptiform changes compared with those whose EEG did not show epileptiform changes (*χ*^2^ = 8.93, d.f. 1, *P* = 0.002). No statistically significant inter-group difference in the rate of psychiatric disorder was detected, according to epilepsy variables such as (a) active (seizure within the past 12 months) versus non-active epilepsy, (b) single versus multiple seizures, (c) generalised tonic-clonic versus focal versus absence seizures, (d) seizure duration for <10 years versus ≥10 years and (e) mono-pharmacy versus polypharmacy of anti-epileptic medication; 30% of those with mild intellectual disabilities and 10% of those with severe intellectual disabilities had a psychiatric diagnosis (*P* = 0.009)
Deb, 1995^42^/UK	Matched controlled study	100 with intellectual disabilities and epilepsy (37% with mild, 23% with moderate and 40% with severe intellectual disabilities). Comparison between 43 with intellectual disabilities and epilepsy and 43 with intellectual disabilities only	20–77	ICD-9 criteria: IQ (mild, 70–50, moderate 49–35, severe <35)	At least three epileptic seizures in two years, according to Gunn and Fenton's (1969) operational definition^48^	DSM-III-R psychiatric diagnosis based on case notes information and family carer interviews (*n* = 100), and SAP for personality disorder diagnosis (*n* = 60)	*χ*^2^	No statistically significant inter-group difference in the rate of psychiatric disorders in participants with generalised epileptiform EEG change (4/12, 33%) compared with focal EEG change (3/18, 17%). No statistically significant inter-group difference in the rate of personality disorders in participants with generalised epileptiform EEG change (3/12, 28%) compared with focal EEG change (8/18, 45%). Participants with epileptiform EEG changes showed no statistically significant difference in the rate of psychiatric disorders (8/43, 19%) when compared with the non-epilepsy control group (15/43, 37%). A statistically non-significant inter-group difference in the rate of psychiatric disorders and personality disorders between those with epileptiform (8/43, 19% for psychiatric disorders; 15/43, 35% for personality disorders, respectively) as opposed to excessive background slowing (16/48, 33% for psychiatric disorders; 13/48, 28% for personality disorders, respectively) in the EEG
Lund, 1985^32^/Denmark	Unmatched controlled study	302 with intellectual disabilities including 55 with epilepsy	>20	See Table 1	See Table 1	An author-devised psychiatric schedule	*χ*^2^	52% of participants in the active epilepsy group (seizures in the past year) had a psychiatric diagnosis, compared with 26% in the non-epilepsy group (*P* < 0.05)
Ring et al, 2007^58^/UK	Observational study	110 with intellectual disabilities and active epilepsy (at least one seizure in the past 3 months) and 65 non-active epilepsy (no seizures in the past 3 months)	16–72	IQ (mild 50–70, moderate 35–50, severe 20–35, profound <20)	Clinical records	Case notes and carer interviews	*χ*^2^	The rates of both psychosis and depressive disorder were higher in the non-active epilepsy group (15% and 34%, respectively) compared with the active epilepsy group (9% and 22%, respectively). The difference was significant at *P* < 0.05. The active epilepsy group showed lower rates of mild intellectual disabilities (14%), but higher rates of severe intellectual disabilities (65%), compared with the non-active epilepsy group (31% and 46%, respectively). In the non-active epilepsy group, psychosis rates were higher in those with mild intellectual disabilities compared with those with severe intellectual disabilities (35% *v.* 3%), and depression rates were lower in those with mild intellectual disabilities compared with those with severe intellectual disabilities (30% *v.* 41%). The difference was statistically significant (*P* = 0.03). In the active epilepsy group, there was no statistically significant difference between mild and severe intellectual disabilities groups in rates of psychosis (13% *v.* 7%, respectively) and depression (33% *v.* 15%, respectively)
Turkistani, 2004^59^/UK	Retrospective study	108 with intellectual disabilities and epilepsy versus 132 with intellectual disabilities alone, unmatched	Mean 40.3	See Table 2	See Table 2	Case-notes-based ICD-10 diagnosis	*χ*^2^	The rate of depression was significantly higher in the less frequent than frequent seizure group (Fisher's exact test, *P* = 0.01). No such inter-group significant difference was present for psychosis (*P* = 0.29)

SAP, Standardized Assessment of Personality; T-L PBI, Temporal-Lobe Personality Behaviour Inventory; ILAE, International League Against Epilepsy; EEG, electroencephalogram.

**Table 4:** Overall and specific psychiatric disorders in publications from 2010 onward

Reference	Sample, control group, study design	Age, years	Intellectual disability diagnosis	Epilepsy diagnosis	Measures used	Statistical analysis	Results
Arshad et al, 2011^27^/UK	156 with intellectual disabilities and epilepsy (43.6% with mild, 23.1% with moderate and 33.3% with severe intellectual disabilities), and 596 with intellectual disabilities alone (68.5% with mild, 22.3% with moderate and 9.2% with severe intellectual disabilities)	Adults	ICD-10 criteria: mild (F70), moderate (F71) or severe (F72–73)	Active epilepsy: at least one seizure in the past 2 years	ICD-10	*χ*^2^	The severity of intellectual disabilities was significantly associated with epilepsy (*P* < 0.001), with higher rates of severe intellectual disabilities being in the epilepsy group. There was a significantly higher rate of psychiatric disorders in the epilepsy group (67.3%%) compared with the non-epilepsy control group (41.44%) (*χ*^2^ = 33.20, *P* < 0.001). There was a significantly lower rate of schizophrenia and personality disorder in the epilepsy group compared with the non-epilepsy control group (*χ*^2^ = 15.01 and *χ*^2^ = 3.91, *P* < 0.001, respectively). Also, there was a higher rate of depression and anxiety disorder in the non-epilepsy (8.6% and 7.9%, respectively) than the epilepsy group (7.1% and 4.5%, respectively). However, dementia was marginally more common in the epilepsy group (3.8%) than in the non-epilepsy group (3.5%)
Dunham et al, 2018^29^/UK	1023 with intellectual disabilities intellectual disabilities (unknown percentage of participants with epilepsy) (38.9% with mild, 24.2% with moderate and 18.9% with severe intellectual disabilities)	16–83	ICD-10 criteria	Not mentioned	Psychiatric assessment	Logistic regression	No association between mental ill health and epilepsy in adults with intellectual disabilities (odds ratio 0.80, 95% CI 0.59–1.07)
Fitzgerald et al, 2011^31^/USA	115 with intellectual disabilities and epilepsy (80.1% with profound and 19.9% with severe intellectual disabilities) compared with 206 with intellectual disabilities only (94.8% with profound and 5.2% with severe intellectual disabilities)	20–88	Clinical records	Clinical records	DASH II	MANCOVA followed by ANCOVA and Bonferroni correction (*P* < 0.004)	No statistically significant inter-group difference in DASH II items, apart from mood subscale (*F*(1315) = 8.67, *P* = 0.003). The epilepsy group (mean 2.91, s.d. 3) had significantly higher ratings on the mood subscale than the control group (mean 1.87, s.d. 3.01). A significant difference in intellectual disabilities level was found between epilepsy and non-epilepsy groups (*P* < 0.001) with higher rates of severe intellectual disabilities and lower rates of profound intellectual disabilities in the epilepsy group
Hermans and Evenhuis, 2013^71^/ the Netherlands	990 older adults with intellectual disabilities including 177 with epilepsy	≥50; mean 61.1, s.d. 8.2	Not mentioned	Not mentioned	For anxiety symptoms: ADAMS (anxiety subscale), GAS-ID, HADS-A. For anxiety disorder: PAS-ADD interview	Logistic regression	No significant inter-group difference in anxiety disorders between the epilepsy and non-epilepsy groups. Increased anxiety symptoms were negatively correlated with epilepsy (odds ratio 0.47, 95% CI 0.24–0.92)
McCarron et al, 2014^56^/ Ireland	77 women with Down syndrome including 53 with epilepsy (69 with dementia; 79.2% with moderate and 19.5% with severe intellectual disabilities)	>35	Not mentioned	Questionnaire	Carer interview and case notes	*χ*^2^	Epilepsy was significantly more common in adults with dementia (73.9%, 51/69) compared with those without dementia (25%, 2/8) (*χ*^2^ = 7.995, d.f. 1, *P* = 0.0075)
Reid et al, 2011^36^/UK	1023 with intellectual disabilities including 334 with epilepsy (38.9% with mild, 24.2% with moderate, 18.9% with severe and 18% with profound intellectual disabilities)	>16	IQ and Vineland Adaptive Behaviour Scales	Healthcare records	PAS-ADD	*χ*^2^	4.3% (15/349) of the epilepsy group and 3.5% (23/663) of the non-epilepsy group had an anxiety disorder (*χ*^2^ = 0.435, *P* = 0.510). Level of ability was significantly associated with anxiety disorders (*P* = 0.032), with 61.5% of participants with anxiety disorders having mild levels of intellectual disability, 15.4% having moderate levels of intellectual disability, 12.8% having severe levels of intellectual disability and 10.3% having profound levels of intellectual disability
Smith and Matson, 2010^46^/USA	25 with intellectual disabilities and epilepsy, and a matched control group of 25 with intellectual disabilities alone (96% with profound intellectual disabilities, 4% unspecified)	17–86	DSM-IV-TR criteria	ILAE criteria	ASD-CA	MANOVA and ANOVA	Statistically higher rates of depressive symptoms in the epilepsy group (mean 0.44, s.d. 0.71) compared with the non epilepsy group (mean 0.32, s.d 0.69) (*F* = 3.73, *P* = 0.01). Statistically higher rates of hyperactivity in the epilepsy group (mean 1.56, s.d. 1.66) compared with the non-epilepsy group (mean 1, s.d. 1.41) (*F* = 5.18, *P* = 0.002)
Turky et al, 2011^62^/UK	52 with intellectual disabilities and epilepsy, 52 with intellectual disabilities alone, matched (46.7% with mild-to-moderate and 53.3% with severe-to-profound intellectual disabilities in each group)	17–80	Deficits in adaptive functioning with onset being before the age of 18 years	Medical records	Mini PAS-ADD to make an ICD-10 diagnosis	ANCOVA	A statistically significant higher depressive symptoms score in the epilepsy group (mean 3.33, s.d. 4.15) than the non-epilepsy group (mean 1.67, s.d. 2.88) (*F* = 5.858, *P* = 0.017), and for unspecified disorders including dementia (*F* = 11.107, *P* = 0.001). No significant inter-group differences in symptom scores of (a) anxiety disorder (*F* = 0.605, *P* = 0.438), (b) mania/hypomania (*F* = 0.364, *P* = 0.547), (c) OCD (*F* = 3.261, *P* = 0.073) and (d) psychosis (*F* = 0.111, *P* = 0.739)
Snoeijen-Schouwenaars et al, 2019^37^/ the Netherlands	189 with intellectual disabilities and epilepsy (20.1% with mild, 30.7% with moderate, 29.1% with severe and 20.1% with profound intellectual disabilities)	18–86; mean 47.9, s.d. 15.6	DSM-5 criteria, using standardised instruments	ILAE criteria (2014)	ADAMS (Dutch version)	Regression analysis	None of the epilepsy characteristics was related to depressive symptoms. Having a more severe level of intellectual disabilities was significantly associated with more depressive symptoms (*P* = 0.013). Anxiety level was significantly higher in people with focal seizures (*P* = 0.034). A lower level of anxiety was significantly associated with a high medication load of mood-stabilizing anti-epileptics (carbamazepine, valproic acid and lamotrigine) and a high seizure frequency (*P* = 0.009 and *P* = 0.006, respectively). Anxiety level was not significantly associated with the severity of intellectual disabilities

DASH-II, Diagnostic Assessment for the Severely Handicapped-II; MANCOVA, multivariate analysis of covariance; ANCOVA, analysis of covariance; ADAMS, Anxiety, Depression, And Mood Scale; GAS-ID, Glasgow Anxiety Scale for People with an Intellectual Disability; HADS-A, Hospital Anxiety and Depression Scale, Anxiety Subscale; PAS-ADD, Psychiatric Assessment Schedule for Adults with Developmental Disabilities; ILAE, International League Against Epilepsy; ASD-CA, Autism Spectrum Disorders-Comorbidity for Adults version; MANOVA, multivariate analysis of variance; OCD, obsessive–compulsive disorder.

Of the included studies, 20 were in the UK, four were in Ireland, two were in the USA, two were in the Netherlands and one was in Denmark.

The studies present data from a total sample of 9594 adults with intellectual disabilities, which included 3180 with epilepsy and 6414 without epilepsy.

### Diagnosis

#### Intellectual disabilities

Included studies used different methods to diagnose intellectual disabilities and evaluate severity. Six studies^11,25,30,35–37^ used standardised evaluation of intellectual disabilities with various psychometric tests, such as Wechsler Adult Intelligence Scale^38^ and Vineland Adaptive Behaviour Scales.^39,40^ Six studies referred to ICD criteria for intellectual disabilities diagnosis, with one study^32^ referring to the eighth edition,^41^ one study^42^ referring to the ninth edition^43^ and four studies^27,29,33,44^ referring to the tenth edition.^45^ One study^46^ used the DSM-IV-TR^47^ to diagnose intellectual disabilities.

#### Epilepsy

Epilepsy diagnosis was based on Gunn and Fenton's 1969 operational definition^48^ in four studies.^25,35,42,49^ Five studies^37,44,46,50,51^ referred to the International League Against Epilepsy criteria.^52,53^

#### Psychiatric disorders

The methods used to define and assess psychiatric disorders differed across the selected studies. Seven studies used a retrospective design and collected data from case notes.^50,54–59^ Four studies^27,28,50,59^ assessed psychiatric disorders based on ICD-10 criteria,^45^ and two other studies^25,42^ used the DSM-III-R.^60^

Of the studies that used validated instruments, three^11,30,36^ used the Psychiatric Assessment Schedule for Adults with Developmental Disabilities Checklist (PAS-ADD)^61^ and one^62^ used the short version of the tool (mini PAS-ADD).^63^ Only one study^10^ used a stringent epidemiological methodology by using a three-stage process for diagnosis. In stage 1, the authors screened the sample with the mini PAS-ADD interview.^63^ Those who met caseness criteria according to the mini PAS-ADD interview^63^ were further interviewed in stage 2, using the full PAS-ADD interview.^64^ In stage 3, information from the full PAS-ADD interview was used to make a psychiatric diagnosis according to the ICD-10 criteria.^46^ One study each used the Diagnostic Assessment for the Severely Handicapped-II,^31,65^ the Psychopathology Instrument for Mentally Retarded Adults^26,66^ and the Autism Spectrum Disorders-Comorbidity for Adults.^46,67^

Two studies^35,49^ used the Standardized Assessment of Personality^68^ to evaluate personality disorders, and two studies^44,51^ used the Test for Severe Impairment^69^ and the Down's Syndrome Mental Status Examination^70^ to evaluate dementia. To evaluate anxiety and depressive disorders, two studies^37,71^ used the Anxiety, Depression, And Mood Scale,^72^ with one study^71^ also using the PAS-ADD interview^64^ for diagnosis of anxiety disorders.

### Statistical methods used

Descriptive statistics, *χ*^2^-test, logistic regression analysis and univariate and multivariate regression analyses were used.

### Outcome

#### The overall rate of psychiatric disorders

We identified 11 controlled studies that compared the rate of psychiatric disorder in epilepsy and non-epilepsy groups, seven^10,26,29–32,34^ of which showed no significant inter-group difference between epilepsy and non-epilepsy groups. Two studies^25,28^ showed a significantly higher rate of psychiatric disorders in the non-epilepsy group. One study^33^ reported a significantly higher rate of psychiatric disorders in the epilepsy group, and another showed a higher rate of psychological symptoms (as opposed to psychiatric disorders) in the epilepsy group compared with the non-epilepsy control group^33^ (see Tables 1 and 4). The findings of the studies published since 2010 (see Table 4) are similar to those that were published before 2010.

#### Rates of different types of psychiatric disorders

Tables 2 and 4 present data from 18 studies that reported rates of different types of psychiatric disorders.

Regarding psychotic disorders, two studies^11,57^ showed significantly lower rates of psychosis in the epilepsy group compared with the non-epilepsy control group, whereas two studies^59,62^ did not find a significant inter-group difference in the rate of psychosis and psychotic symptom scores. Two studies^27,28^ showed a significantly lower rate of schizophrenic spectrum disorders in the epilepsy group compared with the non-epilepsy group. One study^25^ found a non-statistically significant higher rate of schizophrenia in the epilepsy group compared with the non-epilepsy control group.

Concerning depressive disorders, four studies^25,27,57,59^ showed lower rates of depression in the epilepsy group compared with the non-epilepsy controls, whereas two studies showed significantly higher scores of depressive symptoms in the epilepsy group.^46,62^

Concerning anxiety disorders, two studies showed higher rates in the epilepsy group compared with the non-epilepsy group. In the first study,^25^ the difference was not statistically significant, and in the second study,^57^ statistical significance was not reported. One study^27^ showed lower rates in the epilepsy group compared with the non-epilepsy control group. Three studies^33,36,62^ showed no statistically significant inter-group difference in anxiety disorders between epilepsy and non-epilepsy groups.

As for dementia, four studies^44,51,54,56^ found a statistically significant association between dementia and epilepsy. All these studies included participants with Down syndrome. One study^55^ reported specifically on the rate of Alzheimer's disease, and found that epilepsy is significantly more common in those with Alzheimer's disease compared with those without the condition. Three other studies^25,27,62^ presented data related to dementia, but not specifically on participants with Down syndrome. In these studies, inter-group differences were not significant.

Regarding personality disorders, two studies^35,49^ showed no significant difference between the epilepsy group and non-epilepsy control group, and one study^27^ showed lower rates of personality disorders in adults with intellectual disabilities and epilepsy compared with the non-epilepsy group; the significance level was not reported.

#### Association between psychiatric disorders and epilepsy-related variables

Association between overall and/or specific types of psychiatric disorders and epilepsy-related variables has been reported in various studies (see Tables 3 and 4).

Four studies investigated the relationship between seizure types such as focal versus generalised seizures, and rates of psychiatric disorders. None reported a significant association.^32,37,50,58^

On the other hand, epileptic activity was significantly associated with overall psychiatric disorders in two studies.^25,30^ One study^32^ showed a significantly higher rate of psychiatric disorders in the active epilepsy group (seizure in the past 12 months) than the non-epilepsy control group. Active epilepsy, characterised by having at least one seizure in the past 3 months^58^ or at least one seizure in the past 12 months,^35^ was respectively associated with lower rates of psychosis and depressive disorders in one study,^58^ and with higher rates of personality disorders in another study.^35^

Two studies investigated electroencephalogram (EEG) activities associated with overall psychiatric disorders. One study showed higher rates of psychiatric disorders in those whose EEG showed epileptiform changes compared with those whose EEG did not show epileptiform changes,^50^ and one study^42^ showed lower rates of psychiatric disorders in those whose EEG showed epileptiform changes compared with those whose EEG showed excessive background slowing activity. One study showed significantly higher rates of personality disorders in those whose EEG showed epileptiform changes compared with those whose EEG showed excessive background slowing.^42^

Regarding seizure frequency, one study^25^ found significantly lower rates of psychiatric disorders in those with frequent seizures compared with a control group of adults with intellectual disabilities alone (no epilepsy). One study^59^ showed a significant positive association between seizure frequency and depression, and one study^37^ found that lower levels of anxiety were significantly associated with a high seizure frequency in adults with intellectual disabilities.

One study^30^ reported a significant association between psychiatric disorders and seizure severity.

Polypharmacy of anti-epileptic medications was significantly associated with less psychiatric disorders compared with the intellectual disabilities only control group,^25^ with a higher score of personality disorders^35^ and lower levels of anxiety^37^ compared with those receiving mono-therapy of anti-epileptic medication.

#### Psychiatric disorders and the level of intellectual disabilities

Higher rates of psychiatric disorders were found in adults with mild-to-moderate intellectual disabilities compared with adults with severe-to-profound intellectual disabilities in four studies.^25,32,36,50^ The same association was found when investigating psychotic disorders in two studies.^25,58^ Concerning depressive disorders, one study^58^ showed lower rates in those with mild intellectual disabilities compared with severe intellectual disabilities in the group of participants with non-active epilepsy. Another study^37^ found that depressive symptoms were associated with more severe levels of intellectual disabilities, but found no association between anxiety symptoms and severity of intellectual disabilities. However, one study^36^ showed that anxiety symptoms were more frequent in adults with mild intellectual disabilities compared with adults with severe intellectual disabilities.

### Meta-analysis

**Fig. 2:**
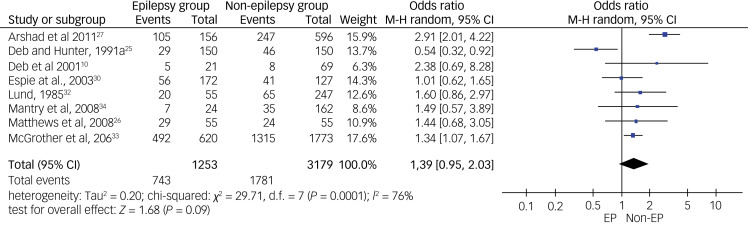
Forest plot of eight studies on overall psychiatric disorders before sensitivity analysis. M-H: Mantel-Haenszel method; EP: Epilepsy.

**Fig. 3:**
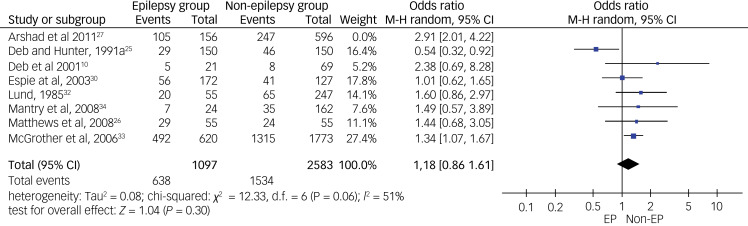
Forest plot of data from seven studies on overall psychiatric disorders after sensitivity analysis. M-H: Mantel-Haenszel method; EP: Epilepsy.

For overall psychiatric disorders, relevant data for meta-analysis were available from only eight out of the 11 controlled studies. Using a random-effects meta-analysis, pooled odds ratio data from these eight studies showed no significant inter-group difference (*P* = 0.09), but the heterogeneity level was high (*I*^2^ = 76%, *P* < 0.01) (see Fig. 2). After the sensitivity check, we have removed data from one study that produced the highest level of heterogeneity and a high risk of bias according to the Cochrane risk of bias tool. This reduced heterogeneity to a borderline moderate level (*I*^2^= 51%, *P* = 0.06). The final meta-analysis from the pooled data from seven studies shows a pooled odds ratio of 1.18 (95% CI 0.86–1.61, *P* = 0.06), using the random-effects model (see Fig. 3). This finding suggests the absence of a statistically significant difference in the rate of overall psychiatric disorders between the epilepsy and non-epilepsy control groups.

**Fig. 4:**
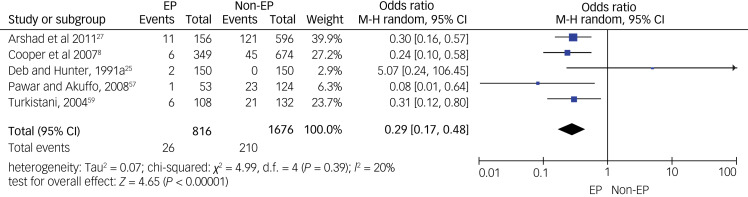
Forest plot of data from five studies on psychotic disorders. M-H: Mantel-Haenszel method; EP: Epilepsy.

**Fig. 5:**
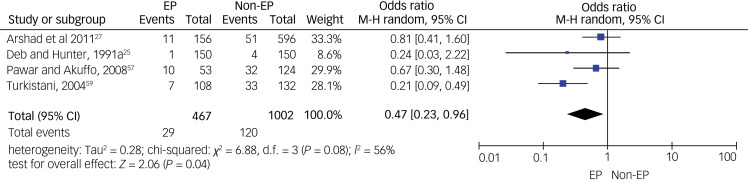
Forest plot of data from four studies on depressive disorders. M-H: Mantel-Haenszel method; EP: Epilepsy.

**Fig. 6:**
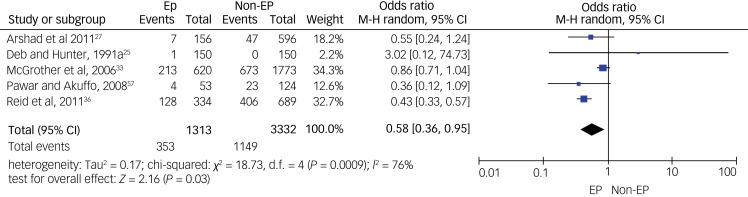
Forest plot of data from five studies on anxiety disorders. M-H: Mantel-Haenszel method; EP: Epilepsy.

As for specific types of psychiatric disorders, we pooled data on psychotic disorders from five studies (see Fig. 4), on depressive disorders from four studies (see Fig. 5) and on anxiety disorders from five studies (see Fig. 6). A meta-analysis of five studies on psychotic disorders showed a statistically significant higher rate in the non-epilepsy control group compared with the epilepsy group, with a small effect size of 0.29 (95% CI 0.17–0.48, *P* < 0.01). The heterogeneity level was low (*I*^2^= 20%, *P* = 0.29). Depressive disorders meta-analysis of four studies showed a statistically significant higher rate in the non-epilepsy control group compared with the epilepsy group, with a moderate effect size of 0.47 (95% CI 0.23–0.96, *P* = 0.04). The heterogeneity level was high, but <60% (*I*^2^= 56%, *P* = 0.08). Meta-analysis of data on anxiety disorders from five studies also showed a significantly higher rate in the non-epilepsy control group compared with the epilepsy group, with a moderate effect size of 0.58 (95% CI 0.36–0.95, *P* = 0.03). The level of heterogeneity between studies was substantial (*I*^2^= 79%, *P* < 0.001).

### Quality control

Five studies were assessed as of high quality, based on the SIGN 50 checklist.

**Fig. 7:**
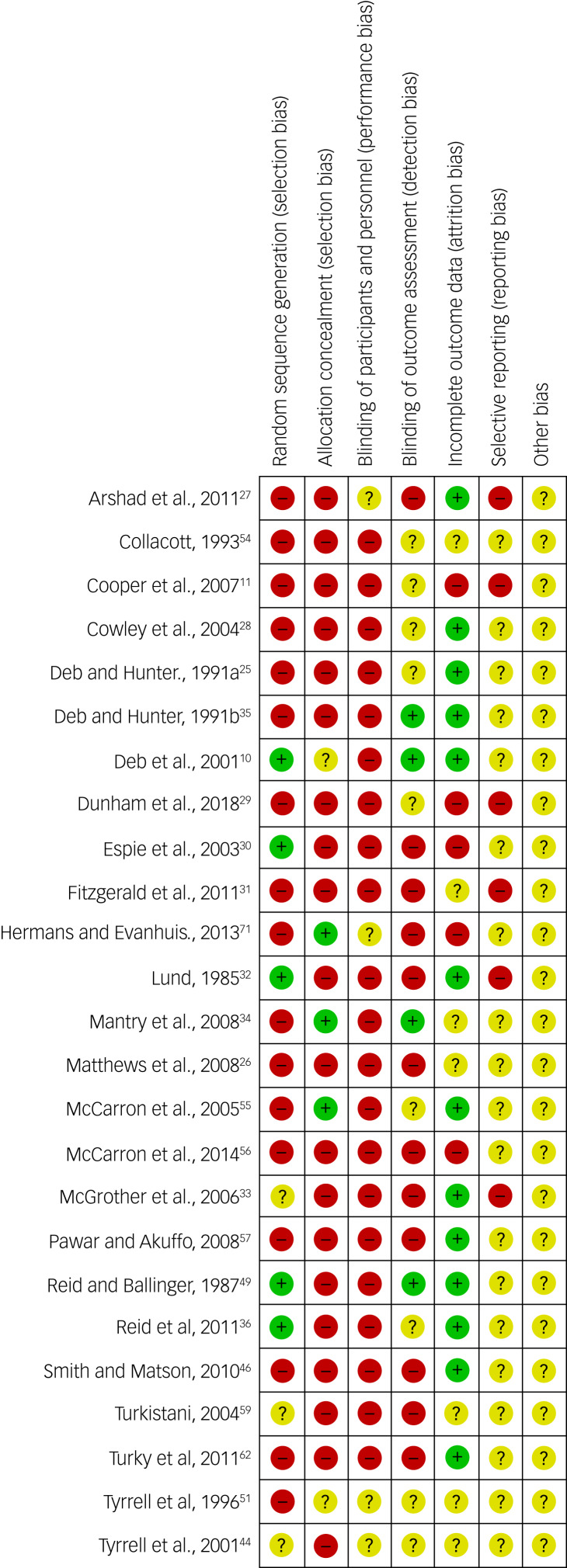
Cochrane risk of bias summary figure for 25 controlled studies. Red with minus sign = high risk of bias; Yellow with exclamation point = unknown risk of biais; Green with plus sign = low risk of bias

Twenty-five controlled studies presenting data on overall and specific psychiatric disorders were assessed with the Cochrane risk of bias tool. A high risk of selection and reporting bias was reported for most studies (see Fig. 7). A summary graph for the risk of bias is presented in Supplementary Appendix 4.

A funnel plot for overall psychiatric disorders, supported by Egger's test, suggests an absence of publication bias (*P* = 0.97). Regarding psychotic disorders, depressive disorders and anxiety disorders, funnel plots and Egger's tests suggest an absence of publication bias (*P* = 0.63, *P* = 0.53 and *P* = 0.72, respectively). Of the included studies in this review, 31% reported receiving external funding.

This systematic review/meta-analysis is of a high standard, based on the AMSTAR 2 checklist (see Supplementary Appendix 2).

## Discussion

The purpose of our systematic review was to explore whether there is an association between epilepsy and psychiatric disorders in adults with intellectual disabilities. We included 29 articles that met eligibility criteria.

A previous systematic review^15^ included only two studies specifically on psychiatric disorders in people with intellectual disabilities. They missed several important studies that we have included in the current systematic review. They did not carry out a meta-analysis. We have included a much higher number of participants within the included studies (*N* = 9594) compared with the previous systematic review (*N* = 7742).^15^

### The overall rate of psychiatric disorders

For meta-analysis, it was possible to pool data on the overall rate of psychiatric disorders from eight out of 11 controlled studies. After sensitivity analysis, we excluded data from one study that produced a high heterogeneity. A meta-analysis of the pooled data from the remaining seven studies did not show any significant inter-group difference in the rate of overall psychiatric disorders between the epilepsy and non-epilepsy groups. This finding is similar to that of the previous systematic review.^15^ A funnel plot and Egger's test showed no publication bias among the included studies. The recent publications did not show any different findings from the ones published before 2010.

To our knowledge, this is the first meta-analysis comparing the rates of psychiatric disorders in epilepsy and non-epilepsy groups of adults with intellectual disabilities. No statistically significant inter-group difference was observed. However, this finding needs to be interpreted with caution, as the included studies are heterogeneous in terms of the definition of psychiatric disorders and instruments used to detect them; although individually most studies showed no significant inter-group difference. To minimise heterogeneity, we have used a sensitivity analysis, and because of different study designs, we have used a random-effects analysis. Also, the Cochrane risk of bias tool deemed most studies to be of poor quality. However, it is worth remembering that Cochrane assessment is designed for intervention studies, particularly randomised controlled trials, and none of the included studies were an intervention study. The main problem with the included studies is that apart from two, none included a matched control group for comparison. Of the two that included a matched control group, one showed a significantly higher rate of psychiatric disorders in the non-epilepsy group, and the other did not show any statistically significant inter-group difference.

Many factors affect the rate of psychiatric disorders in adults with epilepsy, including (a) underlying brain damage, such as the location and severity of any deformity, tumour or abnormal electrical discharge in the brain; (b) epilepsy-related factors, such as certain epileptic syndromes and genetic syndromes, are prone to lead to more psychopathology; (c) seizure-related factors, such as the severity, type and frequency of seizures; (d) anti-epileptic medication-related factors, such as the adverse effects of certain anti-epileptic medications and drug–drug interactions; and (e) psychosocial factors, such as loss of occupation, financial problems, lack of support and locus of control being outside the person so that the person does not have any control over the timing of seizure.^4^ Included studies did not control for these confounding factors. Therefore, it is difficult to know the weighted influence of these factors on the rate of psychiatric disorders reported in different studies. Where data were available, the studies showed a higher rate of psychiatric disorders among adults with less severe intellectual disabilities. This may reflect the fact that psychiatric disorder is difficult to diagnose among adults with more severe intellectual disabilities.^73^

### Psychotic disorders

Our meta-analysis showed a significantly higher rate of psychosis among the non-epilepsy group compared with the epilepsy group. Psychosis is significantly more prevalent in adults with intellectual disabilities compared with the general population,^73^ possibly because of common aetiology involving genetics and environmental factors.^74^ For example, velocardiofacial (22q11.2 deletion) syndrome, a genetic disorder that causes intellectual disabilities, is associated with a high rate of psychosis.^75^ Similarly, the maternal obstetric complication is a common risk factor for both intellectual disabilities and psychosis.^76^ Psychosis is impossible to diagnose among adults with severe and profound intellectual disabilities.^14^ Symptoms of psychosis may be different when epilepsy is present. For example, some suggested that delusions and hallucinations may be unusual and atypical in the general population when associated with epilepsy.^77^ If that is the case, it would be even more difficult to detect psychosis in some adults with intellectual disabilities in the presence of epilepsy. A higher rate of psychosis was associated with a milder form of intellectual disabilities. This may reflect the fact that psychosis is difficult to diagnose among adults with more severe intellectual disabilities.^73^ There may be several reasons for this. For example, the neuronal networks necessary for the production of psychotic symptoms may be damaged or may not exist in adults with severe intellectual disabilities. It is not possible to assess reality-testing reliably in adults with severe intellectual disabilities. There are no valid tools available to diagnose psychosis in an adult with severe or profound intellectual disabilities.

### Depressive disorders

Our meta-analysis showed a significantly lower rate of depressive disorders in the epilepsy group compared with non-epilepsy group, with a small effect size. Many psychosocial factors that are associated with epilepsy can precipitate depression, yet none of the included studies controlled for these potential confounding variables. The rate of depression could have been affected by the use of mood-stabilising anti-epileptics among some of the participants. Some studies suggested that depression may have an atypical manifestation in epilepsy.^78^ If that is the case, it would be even more difficult to diagnose depression in many adults with intellectual disabilities. Where data were available, depression was shown to be more common among adults with a severe intellectual disabilities. This may reflect the fact that depressive disorders may be diagnosed relatively easily among those with severe intellectual disabilities, as the diagnosis is more dependent on observable symptoms, such as sleep disorder and change in appetite.^73^

### Anxiety disorders

Our meta-analysis has shown a significantly lower rate of anxiety disorders in the epilepsy group with intellectual disabilities compared with the non-epilepsy group with intellectual disabilities, although the effect size was small. Therefore, this difference may not be clinically significant. However, some anti-epileptic medications may improve anxiety symptoms.^37,79^

Anxiety is a common symptom in adults with intellectual disabilities. Any change in routine is likely to cause anxiety in this population. The subjective feelings of anxiety, such as palpitation, a sinking feeling and ‘butterflies in the stomach’, are difficult to detect in many adults with intellectual disabilities who cannot communicate their feelings and thoughts. Anxiety may manifest as a problem behaviour in adults with intellectual disabilities, and therefore is not diagnosed as a psychiatric disorder.^14,73^ It is difficult to draw any definitive conclusion from this finding because the number of studies included in the meta-analysis is small, and each included a small number of participants.

### Personality disorders

We included three studies on personality disorder, none of which showed any statistically significant inter-group difference. Diagnosis of personality disorder is controversial among adults with intellectual disabilities.^18^ The relationship between personality disorder and epilepsy is controversial, and standard assessment tools, such as Minnesota Multiphasic Personality Inventory,^80^ have been criticised for not being the right instrument to detect personality disorders in adults with epilepsy in the general population.^81^ As a result, Bear and Fedio^82^ have proposed a specific personality trait related to focal epilepsy of temporal lobe origin. Therefore, the studies included in this review may not be reliable in terms of the validity of personality disorder diagnosis.

### Dementia

All five included studies on dementia showed a statistically significant higher rate of dementia in the epilepsy group compared with the non-epilepsy group. Epilepsy is a common feature of dementia, particularly in the late stage.^83^ All five studies included adults with Down syndrome. People with Down syndrome are particularly prone to developing dementia, and the age at onset is earlier than the general population.^84^ Therefore, the significant association between dementia and epilepsy found in this review is not unexpected. However, the positive association between epilepsy and dementia seems specific to people with Down syndrome and older adults with intellectual disabilities. However, it is worth keeping in mind that one may expect cognitive decline in patients with severe uncontrolled seizures, as a result of ongoing seizure activities and/or head trauma sustained during the seizures.

### Epilepsy-related factors

Included studies that assessed epilepsy-related factors, such as seizure types, active versus non-active epilepsy, seizure frequency/severity, epileptiform changes in the EEG and polypharmacy of anti-epileptic medications, did not find a clear association between the rate of psychiatric disorders and these variables. Subgroup comparisons do not provide adequate power to detect a clinically significant difference because of the small number of participants involved in each subgroup and the lack of control groups. It will be necessary to conduct a larger randomised controlled trial to recruit a reasonable number of participants in each subgroup, to provide adequate power to detect clinically significant inter-group differences.

### Clinical implications

The clinical implications of our findings are manyfold. First, it is not known whether this association is causally related. The underlying brain damage in adults with severe and profound intellectual disabilities, and psychosocial factors in adults with mild intellectual disabilities, may be stronger determinants of psychiatric disorders in adults with intellectual disabilities than epilepsy *per se*.^29^ Some anti-epileptic medications, such as topiramate, may produce psychotic symptoms.^79^ Others, such as lamotrigine and sodium valproate, may have a protective effect against affective disorders. The relationship between anxiety and epilepsy is complex. Although anxiety may be a presenting symptom during the prodrome, pre-ictal, ictal and post-ictal phase, particularly in focal seizures of temporal or frontal lobe origin, anxiety can also precipitate an epileptic seizure. Although the peri-ictal manifestation of psychiatric symptoms is more common, this has not been assessed in the studies included in this review. Sometimes an improvement in seizure control may worsen mental state in adults with intellectual disabilities, but the opposite may also be the case.^81^ The use of antipsychotic medication is common in this population,^85^ which itself may lead to psychiatric symptoms or precipitate seizures. As psychiatric symptoms are precipitated by a complex interaction between internal and external factors, a thorough multi-agency assessment of mental state, using a biopsychosocial model, is essential for the appropriate management of psychiatric disorders and epilepsy in adults with intellectual disabilities.

### Strengths

There are several strengths to our study. We have conducted a systematic review and meta-analysis on psychiatric disorders in an adult population of people with intellectual disabilities and epilepsy, which has not previously been done. Our review received a high score on AMSTAR 2 quality control check for systematic reviews, as we have complied with all their requirements (see Supplementary Appendix 2). Further, we have assessed the risk of bias with SIGN 50 and the Cochrane risk of a bias tool, and included a comprehensive Cochrane risk of bias graph (see Supplementary Appendix 4) and figure (see Fig. 7), which was not done by the other systematic review. Our systematic review has been registered with the well-established PROSPERO database for our protocol to be available for public scrutiny. Finally, we carried out an extensive hand-search of journals in the field of epilepsy and intellectual disabilities, along with rigorous cross-referencing.

### Limitations

There are several limitations to our study. Although a rigorous literature search method was used, it is still possible to have missed some relevant papers. Also, grey literature and abstracts only were excluded, as they would not fit our eligibility criteria and risk of bias assessment.

It is difficult to pool data for meta-analysis from such heterogeneous studies, although individually most of them showed no association between epilepsy and psychiatric disorders in adults with an intellectual disabilities. Although our sensitivity analysis reduced the heterogeneity to an acceptable level, the fact remains that many different instruments and definitions were used for psychiatric diagnosis in included studies. Diagnostic classification systems have changed over time, and different studies have used differing criteria for diagnosing intellectual disabilities and psychiatric disorders. There is a high level of bias caused by the absence of an appropriately matched control group in most included studies. Therefore, to draw a definitive conclusion about the relationship between psychiatric disorders and epilepsy in adults with an intellectual disabilities, it is necessary to carry out more methodologically sound studies in future, using appropriately matched control groups and standardised instruments to detect and define psychiatric disorders in this population.

## Data Availability

Data availability is not applicable to this article as no new data were created or analysed in this study.

## Supplementary material

For supplementary material accompanying this paper visit http://dx.doi.org/10.1192/bjo.2021.55. click here to view supplementary material
