# Supplementary material for: Association between epilepsy and psychiatric disorders in adults with intellectual disabilities: systematic review and meta-analysis
Source: BJPsych Open. 2021 May 3;7(3):e95. doi: 10.1192/bjo.2021.55 (PMC8142548; doi:10.1192/bjo.2021.55)

Appendix 1: Data extraction Form

Appendix 2: AMSTAR 2 checklist

Appendix 3: List of excluded studies with reasons

Appendix 4: Cochrane Risk of bias summary graph

**Appendix 1: Data extraction form.**

# Data extraction form – adapted from Cochrane Collaboration

## Notes on using data extraction form:

- Be consistent in the order and style you use to describe the information for each report.
- Record any missing information as unclear or not described, to make it clear that the information was not found in the study report(s), not that you forgot to extract it.
- Include any instructions and decision rules on the data collection form, or in an accompanying document. It is important to practice using the form and give training to any other authors using the form.

# General Information

| Date form completed *(dd/mm/yyyy)* |  |
| --- | --- |
| Name/ID of person extracting data |  |
| Contact details of person extracting data |  |
| Reference citation (full citation) |  |
| Study author contact details (Email) |  |
| Publication type *(e.g. full report, abstract, letter)* |  |
| Notes: | |

Study details

| Study Name |  |
| --- | --- |
| Author |  |
| Year of publication |  |
| Journal Name |  |
| Volume, Issue, Page |  |
| Status (published, ongoing etc) |  |
| Notes | |

**DO NOT PROCEED IF STUDY EXCLUDED FROM REVIEW**

# Characteristics of included studies

## Participants

|  | Description  *Include comparative information for each intervention or comparison group if available* | | Location in text or source *(pg & ¶/fig/table/other)* |
| --- | --- | --- | --- |
| Population description *(from which study participants are drawn)* |  | |  |
| Setting *(e.g. intensive care unit, service providers, institutions, day care centre etc)* |  | |  |
| Method of recruitment of participants *(e.g. phone, mail, clinic patients)* |  | |  |
| Informed consent obtained | Yes No Unclear |  |  |
| Study Group | Age of participants |  |  |
|  | Number of participants  by sex | M:  W: |  |
|  | Type of epilepsy (e.g. high frequency: 20, low frequency:18) |  |  |
|  | Type of pharmacological regime |  |  |
|  | Severity of intellectual  disability |  |  |
|  | IQ (if reported) |  |  |
|  | Co-morbidities (physical and other) |  |  |
| Control Group | Age of participants |  |  |
|  | Number of participants  by sex | M:  W: |  |
|  | Type of epilepsy (e.g. high frequency, low frequency or type of seizure etc.) |  |  |
|  | Type of pharmacological regime |  |  |
|  | IQ (if reported) |  |  |
|  | Co-morbidities (physical and other) |  |  |
| State different types of psychiatric disorders identified |  | |  |
| Notes: | | | |

## Methods

|  | **Descriptions as stated in report/paper** | **Location in text or source** *(pg & ¶/fig/table/other)* |
| --- | --- | --- |
| **Aim of study** *(e.g. efficacy, equivalence, pragmatic)* |  |  |
| **Design** *(e.g. parallel, crossover, non-RCT, controlled study)* |  |  |
| **Sampling technique (e.g. random)** |  |  |
| Method of establishing Diagnosis | For psychiatric disorders:  For ID: |  |

## Outcomes

*Copy and paste table for each outcome.*

**Outcome 1**

|  | Description as stated in report/paper | | | | | | | | | Location in text or source *(pg & ¶/fig/table/other)* |
| --- | --- | --- | --- | --- | --- | --- | --- | --- | --- | --- |
| Primary outcome if dichotomous (e.g. %) | Number of psychiatric disorders (Epilepsy group) | Total number of participants (N; Epilepsy group) | | Number of psychiatric disorders (non-epilepsy group) | | | Total control number of participants  (N; non-epilepsy group) | | |  |
|  |  |  | |  | | |  | | |  |
| Primary outcome if continuous | Mean (Epilepsy; EP group) | SD (EP) | N (EP) | | | Mean  (non-epilepsy group) | | SD  (non-epilepsy group) | N |  |
|  |  |  |  | | |  | |  |  |  |
| Statistical methods used and appropriateness of these *(e.g. proportion, %, risk ratio, odds ratio)* |  | | | | | | | | |  |
| Secondary outcome |  | | | | | | | | |  |
| Number of missing data |  | | | | | | | | |  |
| Reason for missing data |  | | | | | | | | |  |
| Other |  | | | | | | | | |  |
| Is outcome/tool validated? | Yes No Unclear | | | |  | | | | |  |
| Notes: | | | | | | | | | | |

# Other information

|  | **Description as stated in report/paper** | **Location in text or source** *(pg & ¶/fig/table/other)* |
| --- | --- | --- |
| **Key conclusions of study authors** |  |  |
| **References to other relevant studies** |  |  |
| **Correspondence required for further study information** *(from whom, what and when)* |  | |
| **Notes:** | | |

## Other

| Study funding sources *(including role of funders)* |  |  |
| --- | --- | --- |
| Possible conflicts of interest *(for study authors)* |  |  |
| Notes: | | |

**Appendix 2: AMSTAR 2 checklist**


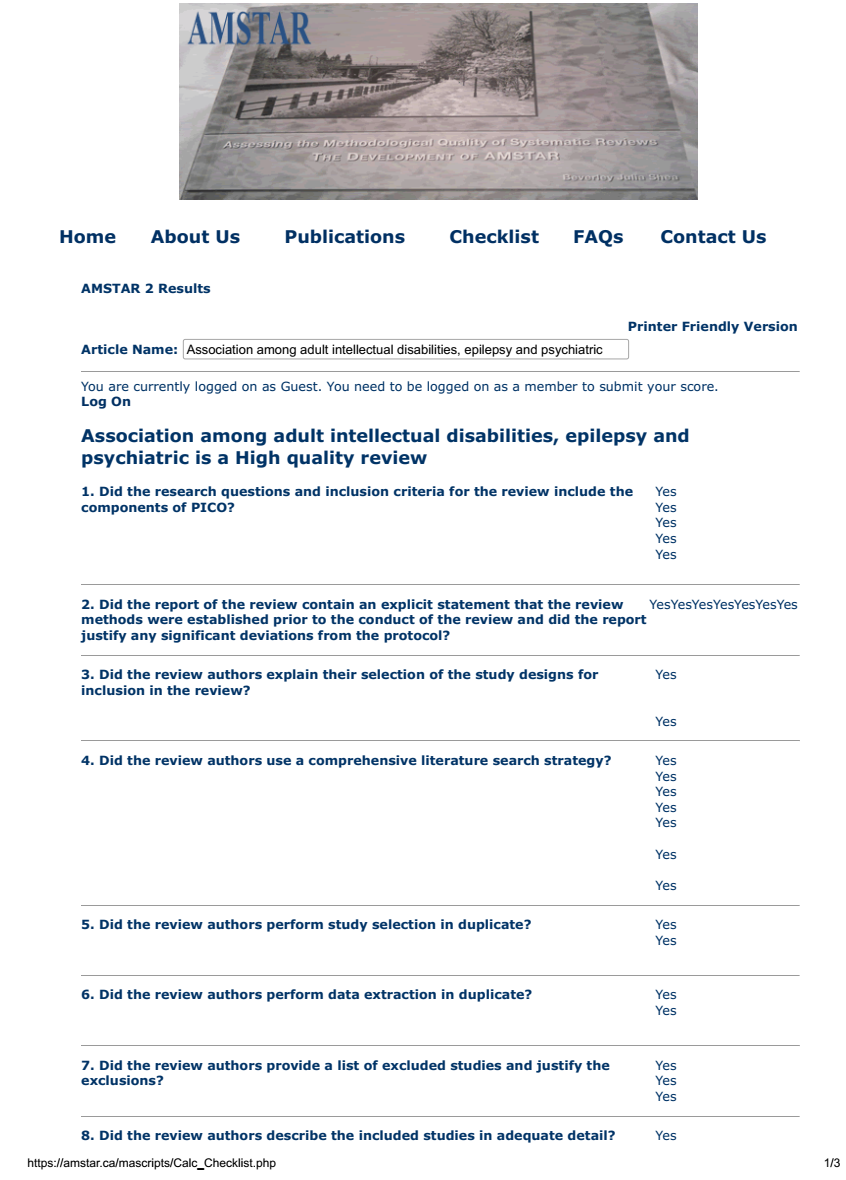


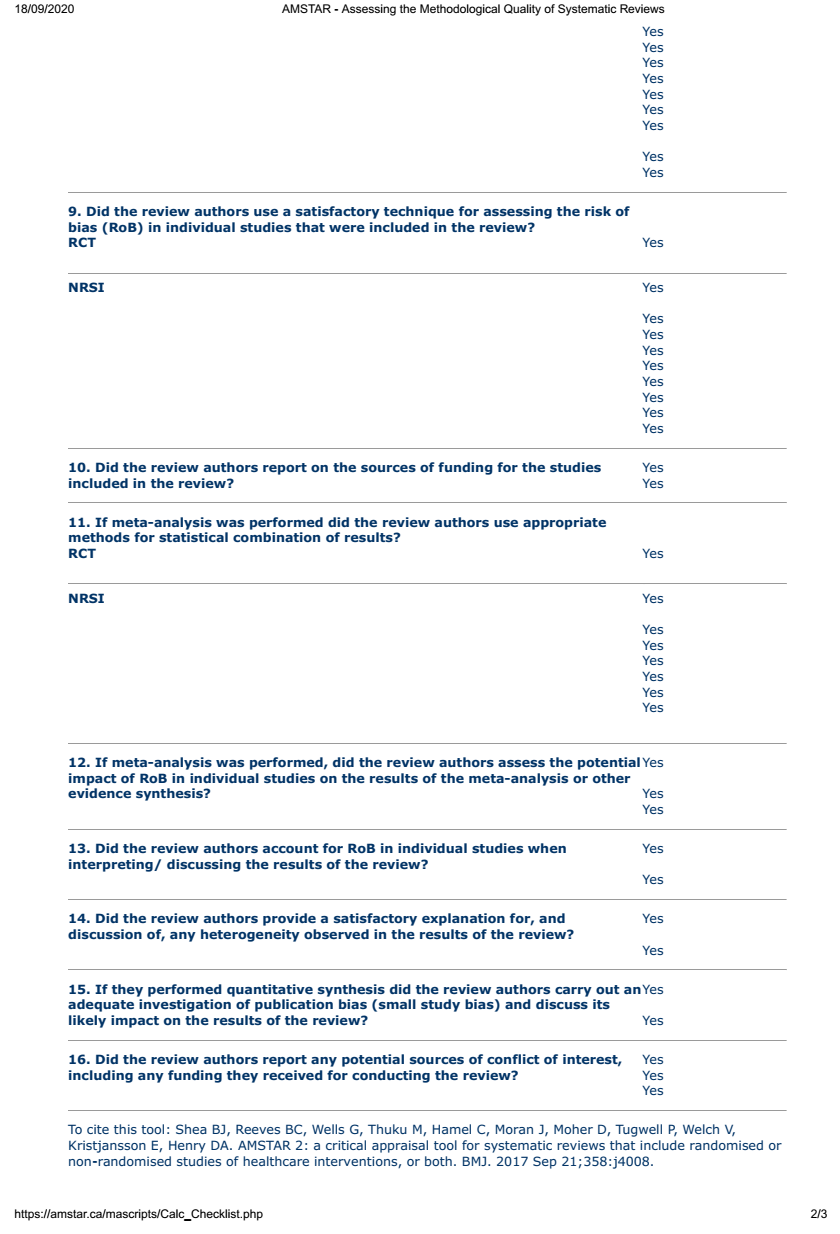


**Appendix 3: List of excluded studies with reasons**

| **Reference** | **Title** | **Reason for exclusion** |
| --- | --- | --- |
| Bhaumik et al., 2008 | Psychiatric service use and psychiatric disorders in adults with intellectual disability | Does not provide data on psychiatric disorders related to epilepsy and ID |
| Deb, 1997 | Mental Disorder in Adults with Mental Retardation and Epilepsy | Presented data on the same cohort as Deb S, Hunter D. Psychopathology of People with Mental Handicap and Epilepsy II: Psychiatric Illness. *Br J Psychiatry* 1991; **159**: 826–30. |
| Doherty et al., 2019 | Eslicarbazepine acetate in epilepsy patients with psychiatric comorbidities and intellectual disability: Clinical practice findings from the Euro-Esli study | Not all participants have ID, no data for psychiatric disorders in ID and epilepsy |
| Endermann, 2015 | Rehabilitation for young adults with epilepsy and mild intellectual disabilities: Results of a prospective study with repeated measurements | Does not provide information on psychiatric disorders in relation to epilepsy and antiepileptic drug related factors |
| Folch et al., 2018 | Health indicators in intellectual developmental disorders: The key findings of the POMONA-ESP project | Does not provide information on psychiatric disorders in relation to epilepsy and antiepileptic drug related factors |
| Lund, 1986 | Behavioural symptoms and autistic psychosis in the mentally retarded adult | Does not provide information on psychiatric disorders in relation to epilepsy and antiepileptic drug related factors |
| McCarron et al., 2017 | A prospective 20-year longitudinal follow-up of dementia in persons with Down syndrome | Same sample as McCarron et al., 2014. |
| McGrother et al., 1996 | Community care for adults with learning disability and their carers: needs and outcomes from the Leicestershire register | Does not provide information on psychiatric disorders in relation to epilepsy and antiepileptic drug related factors |
| McVicker et al., 1994 | Prevalence and associated features of epilepsy in adults with down's syndrome | Does not provide information on psychiatric disorders in relation to epilepsy and antiepileptic drug related factors |
| Mula et al., 2004 | Psychiatric adverse events in patients with epilepsy and learning disabilities taking levetiracetam | Data on psychiatric adverse events of a specific Antiepileptic Medicine (levetiracetam). |
| Zimmermann & Endermann, 2008 | Self–proxy agreement and correlates of health-related quality of life in young adults with epilepsy and mild intellectual disabilities | Does not provide information on psychiatric disorders in relation to epilepsy and antiepileptic drug related factors |
| McCarron et al., 2016 | A prospective 18-year longitudinal follow-up of dementia in persons with Down syndrome | No full text and same sample as McCarron et al., 2014 |
| McCarron et al., 2012 | A prospective fourteen-year longitudinal follow-up of dementia in persons with Down syndrome | No full text |
| Gostason, 1985 | Psychiatric illness among the mentally retarded. A swedish population study | Book |
| Doran et al., 2011 | Seizures are associated with a marked worsening of dementia in Down syndrome | No full text, oral presentation |
| Lott, 2013 | Neurodevelopment and aging in down syndrome | No full text, oral presentation |
| Monetti et al., 2012 | Epilepsy and psychiatric disorders in a “learning disability” patient's series | No full text, oral presentation |
| Deb, 1987 | Effect of folate metabolism on the psychopathology of adults with mental retardation and epilepsy | No data on psychiatric disorders |
| Meins, 1993 | Prevalence and risk factors for depressive disorders in adults with intellectual Disability. | No access to full text |
| Belov & Kazakovtsev,1989 | Psychoses and mental retardation in epilepsy: Clinical aspects, evolution of syndromes, and prognosis | No access to full text |
| Pary, R. (1993). | Mental retardation, mental illness, and seizure diagnosis. | No access to full text |

**Appendix 4: Summary graph of Risk of bias in 25 controlled studies.**


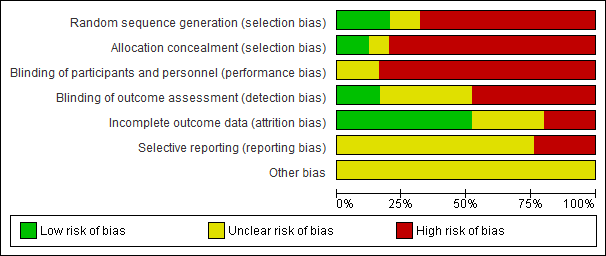

Supplement: Supplementary file 1 [file S2056472421000557sup001.docx]
